# Occurrence of faecal endoparasites in reindeer *(Rangifer tarandus)* in two grazing areas in northern Norway

**DOI:** 10.1186/s13028-021-00578-y

**Published:** 2021-03-23

**Authors:** Lene Idland, Amalie Moen Juul, Ellen Karin Solevåg, Kristoffer Relling Tysnes, Lucy Jane Robertson, Kjersti Selstad Utaaker

**Affiliations:** 1 Parasitology Laboratory, Department of Paraclinical Sciences, Faculty of Veterinary Medicine, Norwegian University of Life Sciences, Adamstuen Campus, Sentrum, PO Box 369, Oslo, 0102 Norway; 2 Faculty of Bioscience and Aquaculture, Nord University, Bodø, Norway

**Keywords:** *Capillaria*, *Dictyocaulus*, *Eimeria*, *Giardia*, *Moniezia*, Nematodirinae, Protostrongylid, *Rangifer tarandus*, Strongylid

## Abstract

**Background:**

Semi-domesticated reindeer represent an important livestock industry and livelihood for a proportion of the human population in northern Fennoscandia. Reindeer husbandry is considered an extensive animal husbandry, where the animals are kept mostly on natural pastures, although sometimes kept in fenced areas for shorter periods. These reindeer may harbour a variety of parasites that may affect animal health and production. The relatively limited close contact between herds and owners gives limited opportunities for diagnosis and treatment of diseases in general. Furthermore, the effects of subclinical parasitism in livestock are commonly expressed as a reduction in productivity rather than clinical disease and mortality. Thus, specific knowledge of endoparasites and parasitic infections in these herds is scarce. This study investigated the occurrence of various endoparasites in reindeer by analysis of a total of 114 faecal samples from winter-slaughtered reindeer from two different grazing areas in Troms and Finnmark, Norway.

**Results:**

Using a McMaster method, a Baermann technique, and a direct immunofluorescent antibody test, the following parasites were identified in the faecal samples with the occurrence data given as percentages: Strongylid eggs (62%), Nematodirinae spp. eggs (24%), *Capillaria* sp. eggs (42%) and *Moniezia* spp. eggs (17%), *Dictyocaulus* spp. larvae (14%) protostrongylid larvae (40%), *Eimera* spp. oocysts (23%), and *Giardia duodenalis* cysts (5%). *Cryptosporidium* oocysts were not detected. Parasite eggs, oocysts, and cysts were quantified.

Molecular analysis revealed *G. duodenalis* sub-assemblage AI, a potentially zoonotic genotype not previously reported in reindeer from this region. Morphological analyses of *Eimeria* oocysts identified two species, *Eimeria mayeri* and *Eimeria rangiferis*, and molecular analyses of the cytochrome C oxidase I (*coi*) gene and 18 s rRNA (*18SSU*) gene of *Eimeria* confirmed the presence of *Eimeria* species that are specific to reindeer.

**Conclusions:**

A high prevalence, but low burden, of endoparasites was detected in samples from these semi-domesticated reindeer. The samples were collected during winter, when adult gastrointestinal parasites usually produce only low numbers of transmission stages. Therefore, together with the low number of samples, detailed and definitive conclusions regarding parasite status of semi-domesticated reindeer are avoided. Nevertheless, these data provide a snapshot overview of parasite occurrence in a semi-domesticated animal group vulnerable to the various environmental changes to which they are exposed.

## Background

Reindeer herding in Fennoscandia can be traced back to the 1500–1600 s. Although this animal husbandry has undergone vast transformations, the most dramatic changes have probably occurred during the last few decades through modernisation of herding by new technology, larger and commercial slaughterhouses, and the commercialisation of reindeer products.

In the Saami reindeer husbandry in Norway, the pasture areas extend from the counties Troms og Finnmark to Innlandet [1]. Around 40% of Norway’s total mainland area is reindeer pasture [2] and constitutes roughly 140,000 km^2^.

The reindeer grazing areas are divided into 6 regional ranges, further divided into 82 grazing districts. In each district, groups of reindeer owners share responsibilities such as herding. These herd groups are called siida or sitje [3].

Troms og Finnmark is the northernmost county in Norway and has the highest number of semi-domesticated reindeer. The number of semi-domesticated reindeer in the Finnmark area in March 2018 was 146,900, which constituted about 70% of the total number of reindeer in Norway. Trendwise, the total number of semi-domesticated reindeer in Norway has decreased by 8% since 2014, mainly due to increased culling [4].

Reindeer are herd animals grazing freely on pastures, which may be shared with other animals and reindeer belonging to more than one owner. Although these pastures constitute large areas of Finnmark county (55,000 km^2^) where the animals can roam freely, there is a theoretical risk of pathogen transmission between herds. This may be associated with feeding, activities such as reindeer racing, outfield industry (hunting, tourism, and international military activity), and trade and transport of live reindeer.

Reindeer mainly graze on natural pastures throughout the year, continuously exposed to environmental challenges (e.g., weather, climate, landscape alterations, and predators).

Although traditionally not considered to represent a challenge to reindeer husbandry, gastrointestinal parasite prevalence and intensity could be expected to increase in the future due to warmer and wetter climates and reductions of areas available for grazing. A large epidemiological study of gastrointestinal parasites in semi-domesticated reindeer calves from Fennoscandia included samples from an area in northern Norway, and the authors highlighted “detailed baseline information about parasitic infections is limited” [5].

The aim of this study was to investigate the occurrence of endoparasites in reindeer from two grazing districts in Troms and Finnmark, Norway, and supply data regarding endoparasite occurrence.

## Methods

### Study area and sample collection

**Fig. 1:**
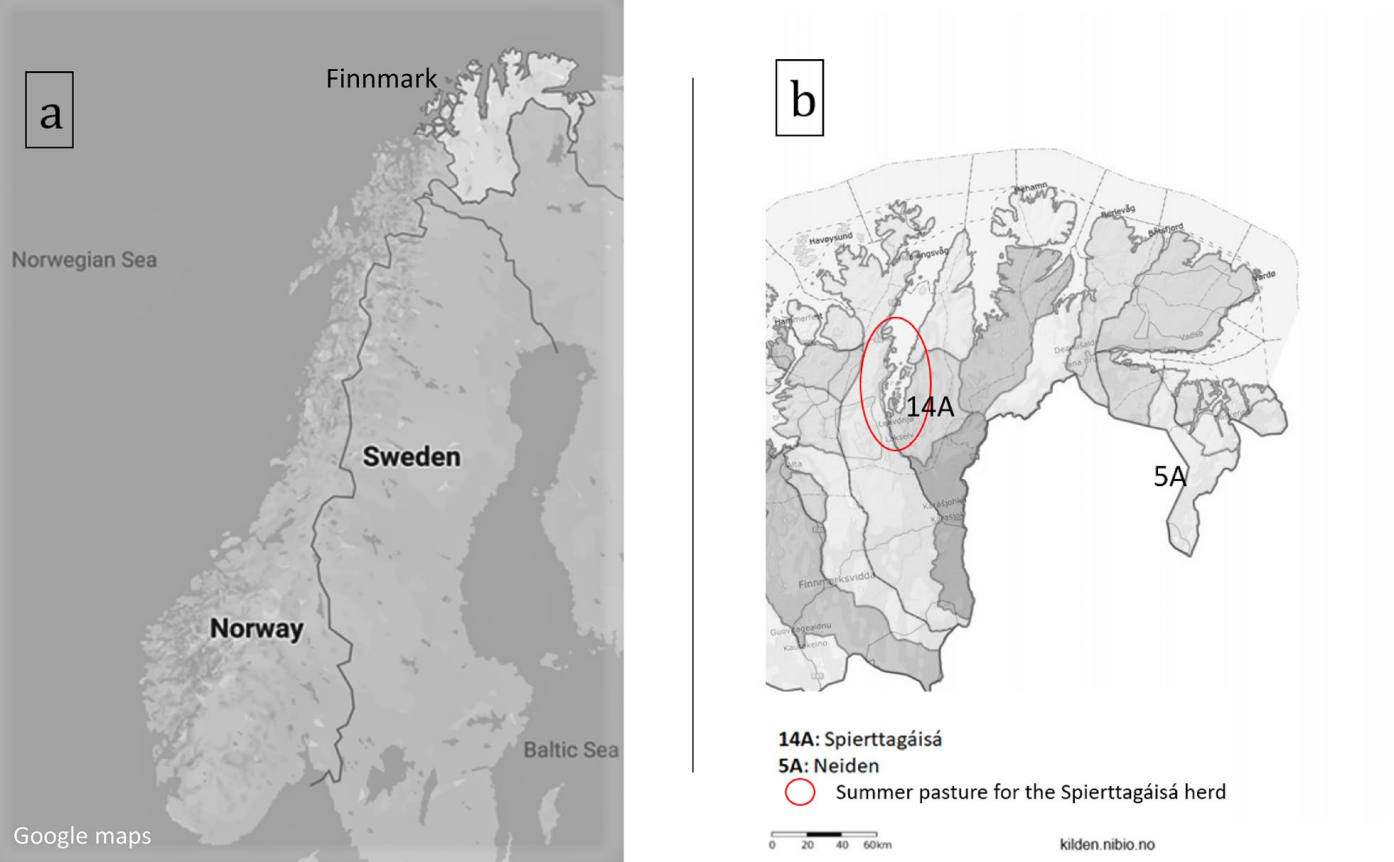
Map of Fennoscandia (**a**) and Finnmark (Troms and Finnmark County, Norway) with grazing areas of reindeer herds sampled and investigated for parasites (**b**)

In October 2017 and January 2018, a total of 114 faecal samples were collected from semi-domesticated reindeer from two different grazing districts in Troms and Finnmark, Norway (Fig. 1a.). Of these, fecal samples from 46 reindeer belonging to the Spierttagáisá district close to Karasjok, although grazed in Porsanger municipality during the summer, and fecal samples from 68 reindeer grazed at Neiden, an area in Sør-Varanger (Fig. 1b). Both areas include sheep pastures, with a relatively low density and number of sheep [6], some cattle farms, and the natural pasture is also shared with an increasing moose population [7]. All samples were collected at the slaughterhouse either in Karasjok or in Kautokeino.

The samples were selected by convenience, and were collected at the slaughterhouse. From both districts, samples were collected from the rectum directly into numbered sample containers along the slaughter line at the station where the intestinal organs were removed. A minimum of 10 g faeces from each animal was collected. The samples from Spierttagáisá were shipped overnight to the laboratory with a cooler, whereas samples from Neiden were cooled outside in the snow (air temperature between − 15 and − 30 °C) for 5 days before overnight transportation to the laboratory in a cool bag. Whether the samples froze during storage in Neiden is unknown. All samples were stored at 4 °C before analysis at the Parasitology Laboratory, Oslo, Norwegian University of Life Sciences (NMBU), and were analysed within 7 days after arrival.

The animals from Spierttagáisá had not received any antihelminthic treatment. Treatment status were unknown regarding the animals from Neiden. In addition to collecting faecal samples, the age group of animals sampled were recorded. All the information was included in a Microsoft Excel database.

## Parasitological procedures

### Modified McMaster flotation test

Faeces (3 g) were homogenized with 57 mL water in a mechanical blender. The suspension was then poured through a metal sieve with a pore diameter of 250 µm for concentration, and divided into two 10 mL tubes, and centrifuged at 1,510×*g* for 3 min. Supernatants were discarded after centrifugation. Sediment from one test tube was resuspended with a saturated NaCl solution (specific gravity 1.18–1.20 g) using a vortex-mixer, and 2 mL of this suspension was transferred to a McMaster counting chamber (Whitlock Universal, Australia). The whole slide was examined at 40 × and 100 × magnification for detection and quantification of helminth eggs and *Eimeria* oocysts.

### Baermann technique

The Baermann technique was used to collect larvae from faecal material for subsequent identification [8]*.* In brief, approximately 5 g of faeces were wrapped in gauze and placed in a funnel connected to a closed tube. The funnel was filled with tepid water and left overnight. Fluid from the stem of the tube was then transferred to 10 mL pointed centrifuge tubes and centrifuged for 5 min at 1,510×*g*. The supernatant was carefully removed until approximately 1 mL was left in the tube, which was examined by microscopy at × 40 and × 10 magnification to identify *Dictyocaulus* spp. and protostrongylid larvae based on morphology. For some samples, a drop of iodine was used to fix and colour the larvae for easier identification. Larvae were not quantified.

### Isolation of *Eimeria* oocysts

An in-lab protocol was used for isolation of *Eimeria* spp. oocysts from positive samples. Attempts were made to isolate from those samples containing > 600 oocysts per gram faeces (a total of nine samples from Neiden and six samples from Spierttagáisá). In brief, faeces were washed in several steps, followed by flotation using 33% ZnSO_4_ solution. Oocysts were collected into 2 mL centrifuge tubes. The isolated oocysts were incubated for about two weeks at room temperature (approximately 22 °C) before being examined for sporulation by microscopy; for this, 10 µL of oocyst suspension was placed on a slide and covered by a coverslip. The slide was examined under × 200 and × 1000 magnification, and structures were measured using the Las X software (Leica Microsystems GmbH, Germany).

### Species identification of *Eimeria* by morphology

To identify the *Eimeria* species, the size and morphology of the isolated oocysts and the sporocysts within were examined by microscopy. Nomarski (differential interference contrast) optics on the same microscope were used to examine morphology, and morphological features (e.g., micropyle, operculum etc.) recorded. An eyepiece graticule and camera imaging system were used to measure the size (length x width) of 20 *Eimeria* oocysts per sample (or fewer if fewer were found), and, for 10 of these oocysts, the size of the sporocyst was also measured. The morphological features were compared with those of other *Eimeria* species for which details have previously been published [9].

### Immunofluorescent antibody test (IFAT)

An immunofluorescence antibody test (IFAT) was used to analyse for the presence of *Giardia* spp*.* cysts and *Cryptosporidium* spp. oocysts on direct faecal smears. Between 5 and 20 µL of homogenized and sieved material was placed on a microscope slide using a plastic bacteriological loop. Slides were airdried before methanol fixation and labelling with 15 µL of FITC-labelled monoclonal antibodies (Mab) against *Cryptosporidium* oocyst walls and *Giardia* cyst walls (Aqua-Glo; Waterborne Inc., NO, USA). The samples were incubated in a humid chamber for 30 min, before removal of excess Mab and the addition of a drop of water and a cover slip. The stained smears were screened by fluorescence microscopy using the following settings: FITC: emission–490 nm, excitation 525 nm, at × 200 and × 400 full magnification. Samples were graded after counting the number of cysts per field of view at × 200 magnification; 1–9 cysts were graded as G + , 10–50 as G +  + , 51–99 as G +  +  + and counts > 100 as G +  +  +  + 

### Immunomagnetic separation (IMS)

*Giardia* cysts were isolated from faecal samples by immunomagnetic separation (IMS) prior to DNA isolation to reduce inhibition during polymerase chain reaction (PCR) from other components in faeces. In brief, 300 µL of concentrated faeces were mixed with 10 μL anti-*Giardia* Dynabeads® (GC-combo; Life Technologies, Carlsbad, CA, USA); 80 µL SurModics StabilZyme®, 20 µL SL Buffer B (GC-combo; Life Technologies, USA), and 100 µL phosphate-buffered saline in a 2 mL microfuge tube. The tubes were rotated for at least 1 h before separation of beads and suspension by magnetic capture, and acid dissociation. The purified sample was stored in a refrigerator at 4 °C until DNA isolation.

### DNA isolation

Samples that were *Eimeria*-positive and/or *Giardia*–positive were selected for molecular characterisation by PCR. First, DNA was isolated from the cysts and oocysts using the PowerSoil® DNA isolation kit (Qiagen, USA) following the manufacturer’s instructions, except that bead-beating was done twice for 60 s at 4 m/s using FastPrep24® 5G (MP Biomedical, USA).

### PCR, electrophoresis, purification of PCR product, and sequencing

Four genes were targeted for genotyping investigations of the isolated *Giardia* cysts; the β-giardin (*bg*) gene [10], the glutamate dehydrogenase (*gdh*) gene [11], the triosephosphate isomerase (*tpi*) gene [12], and the 18 s rRNA (*18SSU*) gene [13]*.* Three genes were targeted for genotyping investigations of the *Eimeria-*positive samples: the cytochrome C oxidase I (*coi*) gene [14], the 18 s rRNA (*18SSU*) gene [15], and the internal transcribed spacer (*its*) gene [16] (Table 1)

### PCR and sequencing

In all cases, the primary PCR consisted of 8.3 μL PCR water, 1 μL forward and 1 μL reverse primers (at a final concentration of 0.1 mM), 0.2 μL BSA (20 mg/L), 12.5 μL of 2 × DreamTaq Green PCR Master Mix (Thermo Scientific) and 2 μL of template DNA. For each PCR, positive control (P101, *G. duodenalis* cysts, human isolate H-3, Assemblage B, Waterborne Inc, LA, USA) and negative control (lab-grade purified water) were included. PCR products were visualized by electrophoresis on 2% agarose gel with SYBR™ Safe DNA Gel Stain (Life Technologies, CA, USA). When positive results were obtained, the DNA amplicons were purified using ExoSAP-IT™ PCR Product Cleanup Reagent (Affymetrix USB, OH, USA) and sent to Macrogen, Netherlands for sequencing in both directions. Sequences from both directions were examined, then assembled and manually corrected by analysis of the chromatograms using the program Geneious™ 10.1.2 software (New Zealand). Sequence comparisons conducted using the National Center for Biotechnology Information Basic Local Alignment Tool (NCBI BLAST, MD, USA). Sequences were submitted to GenBank and have been assigned Accession Numbers (see “Results” section).

### Data description

Microsoft Excel was used to calculate the mean and median of egg/oocyst excretion per g (EPG/OPG) faeces, as well as percentages of positive samples between age groups and areas. Confidence intervals were calculated using the online statistical tool VassarStats [17].

## Results

### Overview

At least 8 parasite genera were detected in the samples. Endoparasites were found in 108 of the 114 samples (94.7%, 95% CI 89–97.6) and 87 of the 114 samples (76.3%, 95% CI 67.7–83.2) contained parasites belonging to more than one genus. Strongylid and *Capillaria* eggs were the most common findings, followed by protostrongylid larvae and *Eimeria* oocysts.

### Occurrence of different parasites

Low levels and high occurrence of parasite eggs were found in the samples from both regions, although the occurrence in animals from Spierttagáisá seemed higher than in than from Neiden for *Dictyocaulus* larvae, *G. duodenalis* cysts, strongylid eggs, and Nematodirinae eggs.

In contrast, the occurrence of *Capillaria* eggs and protostrongylid larvae seemed higher in animals from Neiden. Results are summarised in Tables 2 and 3.

**Table 1:** *Giardia duodenalis* and *Eimeria* spp. primers used in this study

Primer name	Primer sequence	Target	Prod. Size	Reference
*Giardia* primers				
GDHeF	TCAACGTYAAYCGYGGYTTCCGT	bg	432	[10]
GDHiR	GTTRTCCTTGCACATCTCC			
GDHiF	CAGTACAACTCYGCTCTCGG			
G7	AAGCCCGACGACCTCACCCGCAGTGC	gdh	515	[11]
G759	GAGGCCGCCCTGGATCTTCGAGACGAC			
βGiarF	GAACGAGATCGAGGTCCG			
βGiarR	CTCGACGAGCTTCGTTGTT			
AL4303	ATCCGGTCGATCCTGCCG	tpi	255	[12]
AL4305	AGGATCAGGGTTCGACT			
AL4304	CGGTCGATCCTGCCGGA			
AL4306	GGCGGAGGATCAGGGT			
RH11	CATCCGGTCGATCCTGCC	18SSU	292	[13]
RH4	AGTCGAACCCTGATTCTCCGCCAGG			
*Eimeria* primers				
Cocci_COI_For	GGTTCAGGTGTTGGTTGGAC	coi	~ 800	[14]
Cocci_COI_Rev	AATCCAATAACCGCACCAAG			
TK2	GGTTGATCCTGCCAGTAGTC	18SSU	~ 1.800	[15]
ets2	AATCCCAATGAACGCGACTCA			
TK1	AGTAGTCATATGCTTGTCTC			
ITS-For	GCAAAAGTCGTAACACGGTTTCCG	its	200–440	[16]
ITS-Rev	CTGCAATTCACAATGCGTATCGC

**Table 2:** Occurrence of parasites in faecal samples from semi-domesticated reindeer in Spierttagáisá, Finnmark

Grazing area	n positive			Median	Mean	Range
Spierttagáisá	N total = 46 N calves = 34 N adults = 12	% positive	95% CI	epg/opg	epg/opg	epg/opg
Strongylid	39	84.8	71.8–92.43	30	46	10–250
Strongylid (calves)	30	88.2	73.4–95.3	35	45	10–140
Strongylid (adults)	9	75.0	46.8–91.1	30	52	10–250
Nematodirinae sp.	21	45.7	32.2–59.8	40	63	10–280
Nematodirinae sp. (calves)	16	47.1	31.5–63.3	45	73	10–280
Nematodirinae sp. (adults)	5	41.7	19.3–68.1	10	32	10–110
*Capillaria* sp.	1	2.2	0.4–11.3	20	–	–
*Capillaria* sp. (calves)	0	0	0–10.2	–	–	–
*Capillaria* sp. (adults)	1	1	1.5–35.4	20	–	–
*Moniezia* sp.	10	21.7	12.3–35.6	–	–	–
*Moniezia* sp. (calves)	9	26.5	14.6–43.1	–	–	–
*Moniezia* sp. (adults)	1	8.3	1.5–35.4	–	–	–
*Dictyocaulus* sp.	15	32.6	20.1–47.0	–	–	–
*Dictyocaulus* sp. (calves)	14	41.2	26.4–57.8	–	–	–
*Dictyocaulus* sp. (adults)	1	8.3	1.5–35.4	–	–	–
Protostrongylid L1	1	2.2	0.4–11.3	–	–	–
Protostrongylid L1 (calves)	0	0	0–10.2	–	–	–
Protostrongylid L1 (adults)	1	8.3	1.5–35.4	–	–	–
*Eimeria* sp.	7	15.2	7.6–28.2	600	6886	200–45,200
*Eimeria* sp. (calves)	7	20.6	10.4–36.8	500	6886	200–45,200
*Eimeria* sp. (adults)	0	0	0 -30.1	–	–	–
*Giardia duodenalis*	5	10.9	4.7–23.0	–	–	–
*G. duodenalis* (calves)	5	14.7	6.5–30.1	–	–	–
*G. duodenalis* (adults)	0	0	0–30.1	–	–	–
*Cryptosporidium* sp.	0	0	0–7.7	–	–	–
*Cryptosporidium* sp. (calves)	0	0	0–10.2	–	–	–
*Cryptosporidium* sp. (adults)	0	0	0–30.1	–	–	–

**Table 3:** Occurrence of parasites in faecal samples from semi-domesticated reindeer in Neiden, Finnmark

Grazing area	n positive	% positive	95% CI	Median	Mean	Range
Neiden	N total = 68 N calves = 44 N adults = 24			epg/opg	epg/opg	epg/opg
Strongylid	32	47.1	35.7–58.8	10	16	10–40
Strongylid (calves)	22	50.0	35.8–64.2	10	16	10–40
Strongylid (adults)	10	41.7	24.5–61.2	10	15	10–40
*Nematodirinae* sp.	6	8.8	4.1–17.9	20	18	10–30
Nematodirinae sp. (calves)	6	13.6	6.4–26.7	15	18	10–30
Nematodirinae sp. (adults)	0	0	0–13.8	–	–	–
*Capillaria* sp.	47	69.10	57.4–78.8	30	37	10–120
*Capillaria* sp. (calves)	34	77.3	63.0–87.2	35	37	10–120
*Capillaria* sp. (adults)	13	54.2	35.1–72.1	30	37	10–90
*Moniezia* sp.	9	13.2	7.1–23.3	–	–	–
*Moniezia* sp. (calves)	9	20.5	11.2–34.5	–	–	–
*Moniezia* sp. (adults)	0	0	0–13.8	–	–	–
*Dictyocaulus* sp.	1	1.5	0.3–7.9	–	–	–
*Dictyocaulus* sp. (calves)	1	2.3	0.4–11.8	–	–	–
*Dictyocaulus* sp. (adults)	0	0	0–13.8	–	–	–
Protostrongylid L1	45	66.2	54.4–76.3	–	–	–
Protostrongylid L1 (calves)	26	59.1	44.4–72.3	–	–	–
Protostrongylid L1 (adults)	19	79.2	59.5–90.1	–	–	–
*Eimeria* spp.	19	27.9	18.7–39.6	600	1663	200–13,000
*Eimeria* sp. (calves)	19	43.2	29.7–57.8	600	1663	200–13,000
*Eimeria* sp. (adults)	0	0	0–13.8	–	–	–
*Giardia duodenalis*	1	1.5	3.5–7.9	–	–	–
*G, duodenalis* (calves)	1	2.3	0.4–11.8	–	–	–
*G. duodenalis* (adults)	0	0	0–13.8	–	–	–
*Cryptosporidium* sp.	0	0	0–4.7	–	–	–
*Cryptosporidium* sp. (calves)	0	0	0–8.0	–	–	–
*Cryptosporidium* sp. (adults)	0	0	0–13.8	–	–	–

Eggs, (oo)cysts and larvae found in faecal samples from 114 reindeer from two grazing districts in Finnmark. Positive samples, occurrence, 95% confidence intervals, eggs per gram (epg), oocysts per gram (opg), their mean and median and range of counts

## Speciation of *Eimeria* oocysts

### Morphology

*Eimeria* oocysts were not found in samples from adult reindeer. In Neiden and Spierttagáisá, 43% (19/44) and 21% (7/34) of the calves, respectively, were excreting *Eimeria* oocysts.

*Eimeria* oocysts of comparatively large and small sizes were successfully isolated from 5 samples. The average size of the larger oocysts (length x width) (n = 26) was 35.2 × 26.6 µm (range: 31–40 × 25–30 µm), [Standard Deviation; SD: 33.5–37.0– × 24.8–28.4]. Average size (length x width) of sporocysts (n = 20) was 19.9 × 9.9 µm (range: 15–22 × 8.4–10 µm), [SD: 18.8–22.6 × 9.7–10.1 µm]. These measurements are compatible with the species *E. rangiferis* [9].

In one sample from Spierttagáisá, only the smaller *Eimeria* oocysts were detected. The average size (length x width) of these oocysts (n = 6) was 16 × 14.5 µm (range: 15–17 µm × 14–15 µm), [SD: 15.4–16.6] x [SD: 14.0 × 15.0]. These measurements are compatible with the species *E. mayeri* [9]. Screening of several subsamples suggested that this species of *Eimeria* occurred as a monoinfection.

### Molecular analyses

We did not obtain amplification of *Eimeria* DNA using the *its* gene primer set. However, several sequences obtained using the *coi* gene and *18SSU* gene primers were of good quality and showed close genetic relationships with other *Eimeria* species when aligned with sequences in the NCBI GenBank database. Five sequences have been submitted to GenBank.

From the sample containing only oocysts identified as *E. mayeri* by microscopy, the sequence results produced sequences of good quality from both the *coi* gene and *18SSU* gene primers (GenBank Accession Numbers: MK170375.1 (*18SSU*) and MT987642 (*coi*). As several subsample screenings confirmed this was the only *Eimera* species present, the sequence aligning to MT987642, though from another sample, were thus confirmed to belong to this species as well (MT987644). The closest match in GenBank for the *coi* sequences was *Eimeria mephtidis* (91.81% identity) and for *18SSU* it was *Eimeria subspherica* (97.96% identity).

From two samples containing larger oocysts confirmed to belong to *E. rangiferis* the sequence results produced sequences of good quality from the *coi* gene primer (GenBank Accession numbers: MT987643 and MT987645)*.* These sequences were identical to each other, but not to the sequences obtained from the sample containing only *E. mayeri* oocysts*.* Among published sequences in GenBank *Eimeria melogale* was the closest match (92.02% similarity).

### *Giardia* cysts

Overall, *Giardia* cysts were found in 5% (6/114) of the samples collected. One sample was from Neiden, and five were from Spierttagáisá. Of these six positive samples, four had a low number of cysts (+), one had a moderate number of cysts (+ +), and one had a high number of cysts (+ + +).

PCR was attempted for all six positive samples, and positive results were obtained from three of them. Different genetic loci gave the following positive results: *bg* gene 33% (2/6), *gdh* gene 17% (1/6), *18SSU* gene 33% (2/6) and *tpi* gene 33% (2/6).

Of the sequences obtained, all belonged to Assemblage A, sub-assemblage AI. The sequences have been deposited in GenBank under the Accession Numbers: MH155687, MH155689 for the *bg* sequences, MH051905 for the *gdh* sequence, MH04726-MH047247 for the *18SSU*-sequences and MH155689-MH155690 for the *tpi* sequences.

### *Cryptosporidium* sp.

*Cryptosporidium* sp*.* oocysts were not detected in the samples.

## Discussion

The main finding in this study was that a low number and a high prevalence of different endoparasites seem to be common in apparently healthy reindeer from the two areas. The majority of samples were positive for one or more parasite species. Although the sample size and area were limited, the results are consistent with those from earlier and larger studies reporting that semi-domesticated reindeer harbour a high prevalence of subclinical, low-intensity, mixed infections of gastrointestinal parasites [5, 18–21].

Reindeer husbandry represents a traditional and vital production system in Troms and Finnmark county, and any factor that may impose a constraint on their productivity may impact the local economy. Information on endoparasites in semi-domesticated reindeer in Norway is limited, and the results of this study only provide a snapshot overview among a low number of animals from two regions. All animals were slaughter animals that underwent ante- and post-mortem inspections; no manifestations of clinical disease were observed.

From the parasites detected in this study, of particular note is the presence of a zoonotic assemblage of *G. duodenalis.* Such infections have not previously been recorded from semi-domesticated reindeer in Norway.

A range of trichostrongylid nematodes have previously been reported from reindeer [e.g., 22–26, and reindeer are susceptible to infection with strongylids typically found in domestic animals [27]. Nevertheless, the dominant gastrointestinal strongylid species in reindeer has been reported to be the abomasal trichostrongylid *Ostertagia gruehneri* [28], and we assume that this is the species that was found in our study, given that the natural pastures in this study were predominantly grazed by reindeer.

*Ostertagia gruehneri* egg output generally peaks mid-summer or in late summer-autumn [29], and the burden of adult *O. gruehneri* has been found to increase from autumn to spring in Svalbard reindeer [30]. *O. gruehneri* infections have been associated with decreased food intake, loss in body condition and reduced fecundity in reindeer [31]. Egg counts of *O. gruehneri* have been shown to decrease during the winter months, although this decrease followed the reduction in adult nematode burdens in the abomasum and an increase in larval stages in the abomasal mucosa [19]. In this study, the strongylid egg counts were low, which may not reflect the actual abomasal nematode burden, due to the time and logistics of sampling.

Nematodirines are found in the small intestine of mainly young ruminants, and the two species considered specific to reindeer are *Nematodirus tarandi* and *Nematodirella longissemispiculata*. *Nematodirus skrjabini* has also been reported, but this may be a synonym for *N. tarandi* [30]. A recent epidemiological study from Fennoscandia reported a 22.1% prevalence of Nematodirinae eggs in reindeer calves [5], which seems lower than our findings. A survey from four areas in northern Finland [33] only found Nematodirinae spp. in reindeer calves and concluded that reindeer Nematodirinae infection appears similar to that of *Nematodirus* spp. in domestic ruminants, where infected young animals rapidly develop a strong immune response to re-infection.

*Capillaria* sp. is a nematode commonly found in reindeer of all ages. Eggs are shed intermittently, which may underestimate the true prevalence when single samples are examined, and in contrast to *O. gruehneri*, eggs are shed even in mid-winter [20]. A study using consecutive sampling [19] found that 60% of calves in northern Finland shed eggs during the winter, with an age-related difference in egg output. A recent survey [5] found a 9.4% prevalence in calves from both Finland and Norway. The seemingly geographical differences in this study may reflect local establishment of parasite life cycles.

*Moniezia* spp. tapeworms are probably a common parasite of reindeer in Fennoscandia, although reports are scant. Studies from the late 1970s on semi-domesticated reindeer in Reinøya, Troms found an 18% prevalence during late summer [34], an 18% prevalence in calves from Northern Finland was reported in 2008 [35], and a prevalence of 28% was found in Finnish and Norwegian reindeer calves in 2019 [5]. *Moniezia* infections in reindeer are reported to be most common in young animals [36]. The overall impact of *Moniezia* spp. on reindeer in Fennoscandia is yet unknown, though low intensity infections are, as with *Moniezia* infections in domestic ruminants, assumed to be of little clinical importance.

Different species of *Dictyocaulus* have been reported from both wild and domestic ruminants in the Holarctic, with *D. eckerti* considered as the most common lungworm of reindeer [37]. Prevalence data on lungworm infection in semi-domesticated reindeer in Norway are scarce, although a longitudinal study from 1978 to 1979 found a prevalence ranging from 0 to 42% [34], with the highest proportion of positive samples collected in May and June. Pathological changes from lungworm infection inspected at a slaughterhouse during 1978–1980 amounted to less than 1%, but it was noted that this may be a misrepresentation of the prevalence, as animals usually develop immunity with age [22]. In our survey, the occurrence was considerably lower than recent findings from wild reindeer populations in Norway, where prevalences from 28 to 80% were found [38]. Although presumed to have an overall low prevalence in semi-domesticated reindeer, outbreaks of disease due to *Dictyocaulus* spp*.* have been reported in reindeer calves, sometimes with the contribution of opportunistic bacteria, and have caused severe mortality in calves during spring [39].

*Elaphostrongylus rangiferi*, or the meningeal worm of reindeer, is widespread among semi-domesticated reindeer in Norway [40]. Prevalences up to 68% have previously been found in calves [41], and up to 100% in adult reindeer. A recent study on wild reindeer found prevalences up to 89% in some herds [38]. In contrast to the direct life cycle of *Dictyocaulus* spp., *E. rangiferi* requires a gastropod intermediate host to complete its life cycle, and further development inside this intermediate host is temperature dependent. Warm summers have been followed by mass outbreaks of neurological brainworm infection. One study [42] concluded that above-mean summer temperatures facilitate mass development of infective *E. rangiferi* larvae in the gastropod intermediate hosts and thus can cause epidemics of cerebrospinal elaphostrongylosis with high mortalities, especially among calves. In the unusually warm summer of 2018, reindeer herders in Norway reported an outbreak of elaphostrongylosis; one herder apparently lost 70 animals of different ages [43]. Under conditions of predicted climate change, such outbreaks of elaphostrongylosis may increase.

We believe the protostrongylid larvae found in this study belong to the species *E. rangiferi*, as they did not have the shorter length and granulated appearance of *Muellerius capillaris* lungworms. Furthermore, they are unlikely to be *Neostrongylus* spp., *Protostrongylus* spp. and *Cystocaulus* spp., as the larvae found in our samples had the characteristic kinked tail and dorsal spine of *Elaphostrongylus* spp. However, we cannot exclude that at least some of the larvae in our samples could be *Elaphostrongylus alces* or *Vareostrongylus eleguneniensis*, resulting from spillover from the locally increasing moose population, although, based on previous reports, *E. rangiferi* may be the most plausible species in this case.

Eight species of *Eimeria* and one *Isospora* species have been described from reindeer. Five of these are based on oocysts recovered from faeces from reindeer in Russia: *E. arctica, E. mayeri, E. mühlensi, E. tarandina* and *I. rangiferis* [44–46], two are based on oocysts recovered from faeces from reindeer in Iceland; *E. rangiferis* and *E. hreindyria* [9], and a new species, *E. tuttui*, was described from reindeer in Greenland in 2016 [47]. To our knowledge, this study provides the first identified species of *Eimeria* found in semi-domesticated reindeer in Norway. As reindeer from Iceland are descendants of Norwegian reindeer, it is not surprising that they share the same species of this host-specific parasite.

Clinical disease associated with *Eimeria* spp. is not considered common in free–ranging wild reindeer. A study from Finland found the prevalence in semi-domesticated reindeer calves to be 35% [48], whereas a survey of wild reindeer calves from Iceland found a 3.6% prevalence of *Eimeria* oocysts in faeces [25]. Little is known regarding the pathogenicity caused by *Eimeria* infections in reindeer, and, under natural grazing conditions, clinical infections are probably not common [21]. As infection with direct life cycle parasites is favoured by crowding in enclosures, *Eimeria* spp. could be among the parasites that will increase in burden and prevalence in semi-domesticated reindeer if duration and/or frequency of gathering in enclosures (e.g. calving and feeding) increase, and pasture area continue to fragment and decrease.

*G. duodenalis* is a common intestinal parasite found in mammals, which, depending on assemblage, has both anthropozoonotic and zoonotic potential. Assemblages A and B have a wide host spectrum and are further divided into different sub-assemblages based on genetic diversity. Some sub-assemblages are found in both humans and other mammals, giving a strong indication that the genotype is zoonotic. Sub-assemblage AI is preferentially found in animals, AII is more often found in humans, while AIII is exclusively found in animals, particularly wildlife [49]. The detection of assemblage AI in reindeer calf faeces from Neiden and Spierttagáisá indicates a zoonotic potential. Although *G. duodenalis* of sub-assemblages AI and AII have previously been found in wild reindeer and moose in Norway [50], this has not been previously described in semi-domesticated reindeer in Norway.

*Cryptosporidium* spp. have been sporadically detected in reindeer faeces. An epidemiological study from northern Finland and Norway did not detect *Cryptosporidium* spp*.* in semi-domesticated reindeer [51], but infections have been observed in reindeer calves and were associated with diarrhoea [21]. In wild reindeer, *Cryptosporidium* spp. infections are considered uncommon and have not been associated with clinical disease [52]. A cumulative prevalence of 100% *G. duodenalis* cysts and 23% *Cryptosporidium* oocysts found in faeces from semi-domesticated reindeer calves from age 0 to 33 days in a study from Northern Finland [53] demonstrates that these parasites are present and have possibly established in some semi-domesticated reindeer herds.

Pasture parasite development is dependent on environmental factors like temperature and humidity, thus their prevalence is likely to vary throughout the year, being higher during summer and lower during winter [19].

In this study, samples were collected and stored inconsistently. Samples from one area were stored at varying air temperatures (ranging between approximately − 15° and − 30 °C) and this may have affected the results. In addition, samples were collected during late autumn and winter, and previous studies have found that egg output decreases in this period due to changes in the gastrointestinal parasite population structure with fewer adult, egg-laying worms, or a natural regulation of egg production during a season where conditions for free-living stages on pasture are suboptimal [19]. Our results should thus be interpreted with caution and cannot be representative as a true prevalence or burden. The small sample size is also a limitation of the study, although indicating the need for further investigations with more targeted and larger sample sizes.

In this study we found parasites in a high number of samples, but none of the sampled animals shed quantities that are considered to reflect clinical relevance when compared to infections acquired by domestic ruminants kept under more intensive husbandry. Contributing factors to the low intensity of infection can be the time of sampling and low animal or parasite density on feeding grounds and pasture.

During the last decades, the northernmost counties of Norway have experienced an increase in tourism. This has, in turn, increased the local globalisation in this area.

The need for and use of supplementary feeding has increased as countermeasures for difficult winter seasons when food availability is restricted, pasture areas are decreased, and climate changes, as well as abnormal seasonal weather resulting in higher temperatures and increased precipitation making food less accessible when snow turns to rain and then ice [54]. Unstable winters, with mild weather and coastal climates moving further inland, have resulted in unpredictable access to pasture resources for reindeer in winter. We speculate that these changes may increase the need for calving inside protective fences and thus feeding inside these fences.

Supplementary feeding and keeping reindeer in protective fences are also a move away from traditional reindeer herding. Although this may increase tameness and facilitate monitoring of the herd [55], it can also result in higher animal densities and thus greater infection pressure.

As parasitic infections in reindeer and their clinical significance are still largely unknown and reindeer husbandry faces an uncertain future, we hope that the results we obtained from this study may be part of a future reference for further studies of these relatively unexplored areas of parasitology and epidemiology. We also hope it may contribute to a better understanding of the potential hazards these infections may represent for a traditional industry that is vulnerable to seemingly unavoidable future changes.

## Conclusions

We detected a high prevalence, although low burdens, of faecal endoparasites (perhaps reflecting time of sampling) in a limited number of samples from semi-domesticated reindeer in Finnmark, Norway.

With many reindeer harbouring parasites, as shown in this and other studies, changes in weather, climate, and husbandry favourable for parasite accumulation and development could result in exacerbated infection pressure and development of parasitic diseases in semi-domesticated reindeer. Considering the ongoing climate change, there is a clear need to broaden our knowledge regarding the various challenges that reindeer husbandry might face.

## Data Availability

The datasets used and/or analysed during the current study are available from the corresponding author on reasonable request. The sequences obtained have been assigned the following accession numbers: *Giardia duodenalis*: MH155687, MH155689, MH051905, MH04726-MH047247 and MH155689-MH155690. *Eimeria* spp: MK170375.1, MT 987,642, MT987643, MT987644 and MT987645.
